# Supplementary material for: Chemical heterogeneities reveal early rapid cooling of Apollo Troctolite 76535
Source: Nat Commun. 2021 Dec 14;12:7054. doi: 10.1038/s41467-021-26841-4 (PMC8671448; doi:10.1038/s41467-021-26841-4)
Supplement: Supplementary file 1 — Supplementary Materials [file 41467_2021_26841_MOESM1_ESM.pdf]

Chemical heterogeneities reveal early rapid magmatic cooling of Apollo Troctolite 76535

William S. Nelson*, Julia E. Hammer, Thomas Shea, Eric Hellebrand, G. Jeffrey Taylor

Supplementary Materials

Supplementary Figures:

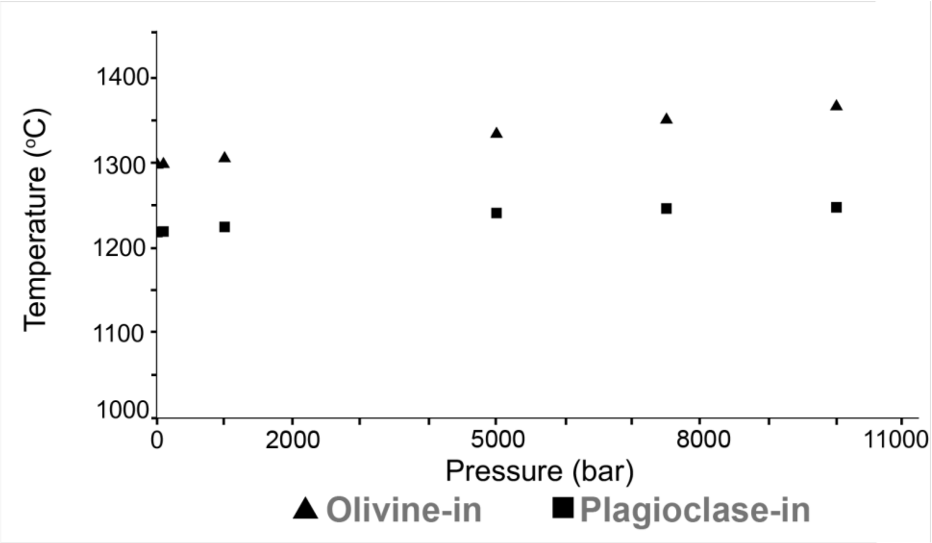

Supplementary Figure 1: Olivine and plagioclase in-temperatures for the composition shown used to define initial temperature for modelling (Methods, Supplementary Table 1).

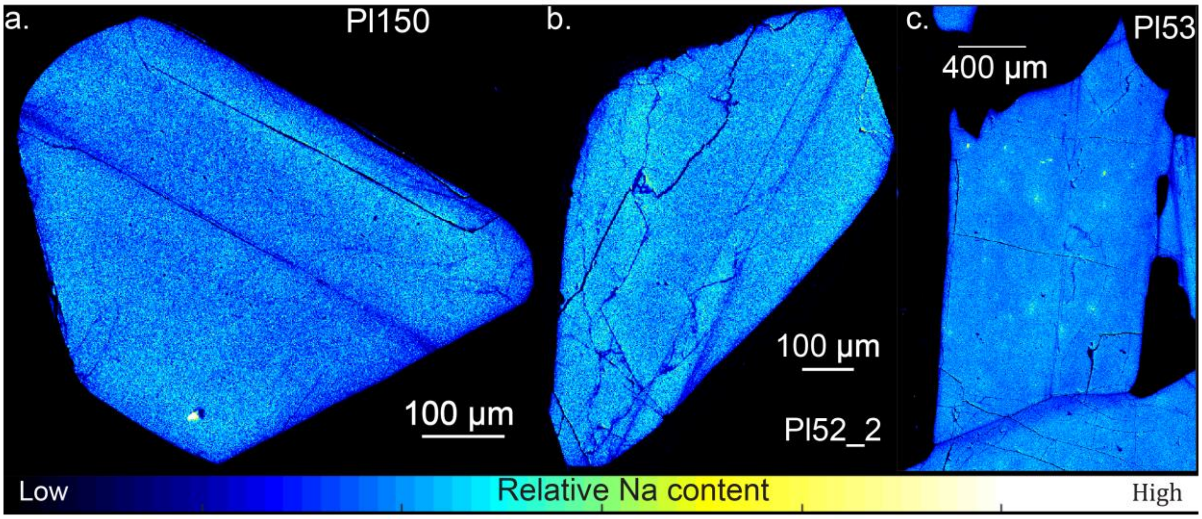

Supplementary Figure 2: X-ray intensity maps of plagioclase grains that were not selected for numerical modeling. a. Plagioclase grain in 150: This grain displays heterogeneities as well as igneous growth facets, but cracks render it unsuitable. b. Another plagioclase in slide ,52. This pervasively cracked grain contains spatially resolvable compositional variation (concentric zoning) that may have initially been coherent but cannot be analyzed numerically. c. This plagioclase grain in slide ,53 lacks detectable compositional zoning. Note the lower Na concentration near the neighboring plagioclase crystal, which suggests that intra-plagioclase boundaries are a pathway for Na migration.

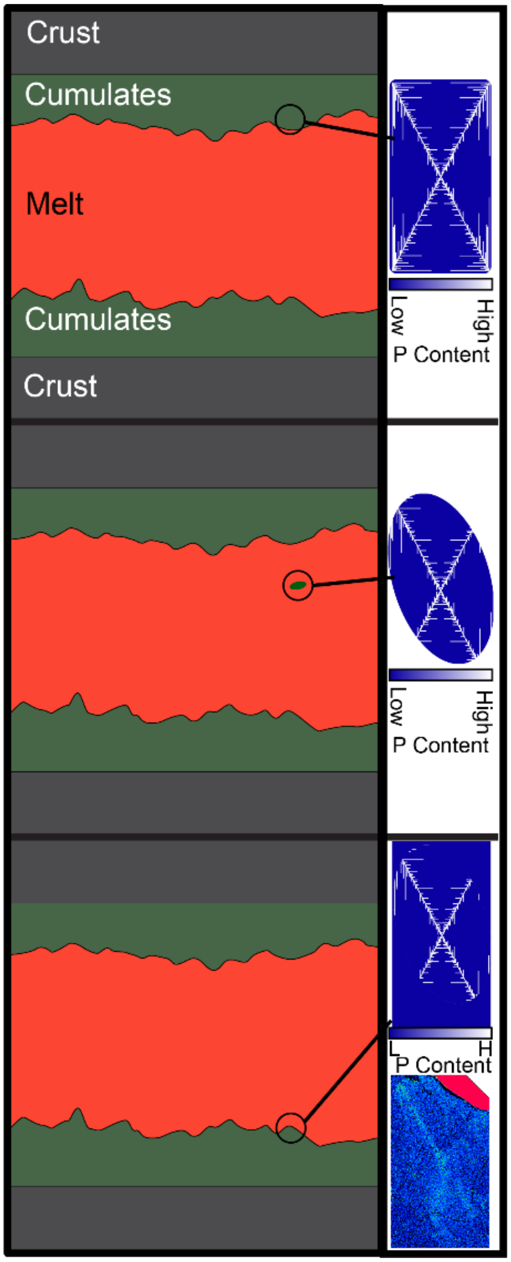

Supplementary Figure 3: Alternative model to generate P-rich lamellae and dissolution bounds. An olivine crystal grows rapidly on the roof of a magma chamber, preserving compositional heterogeneities. The crystal detaches and sinks through the melt-rich center of the chamber. Conditions at the center of the chamber are outside of the olivine stability field, and the crystal partially dissolves. The remaining portion of the grain settles to the bottom of the chamber, where olivine is again stable, and growth resumes. We argue this model is not a plausible explanation for the textural features and compositional variations within the samples we analyzed. The sharpness of the lamellae point to a more rapid cooling history than this scenario can realistically offer. Also, the model only explains the growth-dissolution-growth relationships for crystals that settled through the melt column, as illustrated. We infer that the olivine we observed is of general relevance, rather than a special case. This also points to a mechanism, such as reactive infiltration, that operates on a majority of olivine grains.

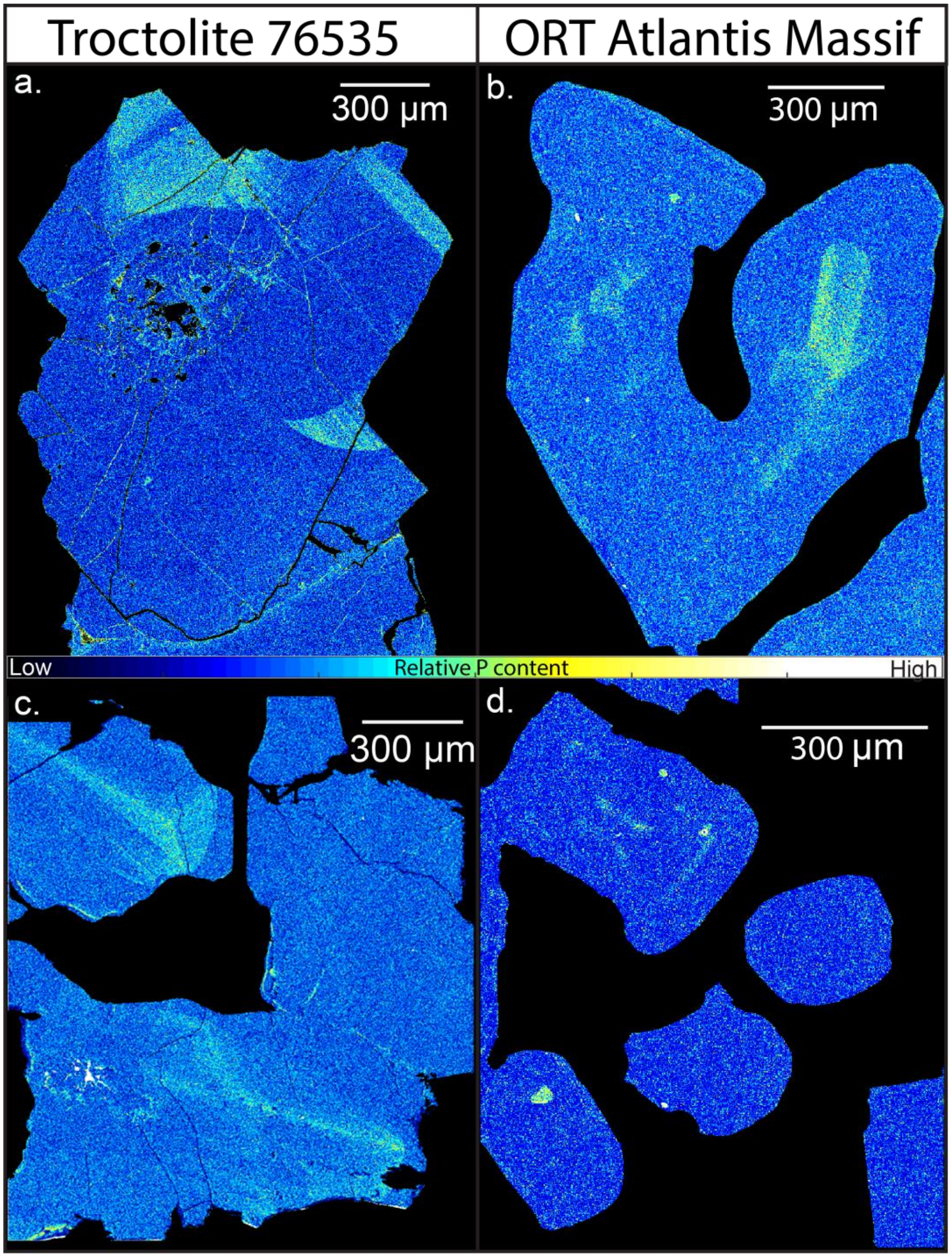

Supplementary Figure 4: Comparison of P rich zoning in troctolite 76535 (a,c) and olivine rich troctolites (b,d) from Atlantis Massif[24,33]*.* Maps only display relative intensity. Actual phosphorus content varies: the maximum P content found in ORT grains was 524 ppm[24]; the observed P contents in 76535 extend above 1000 ppm (Supplementary Table 7)

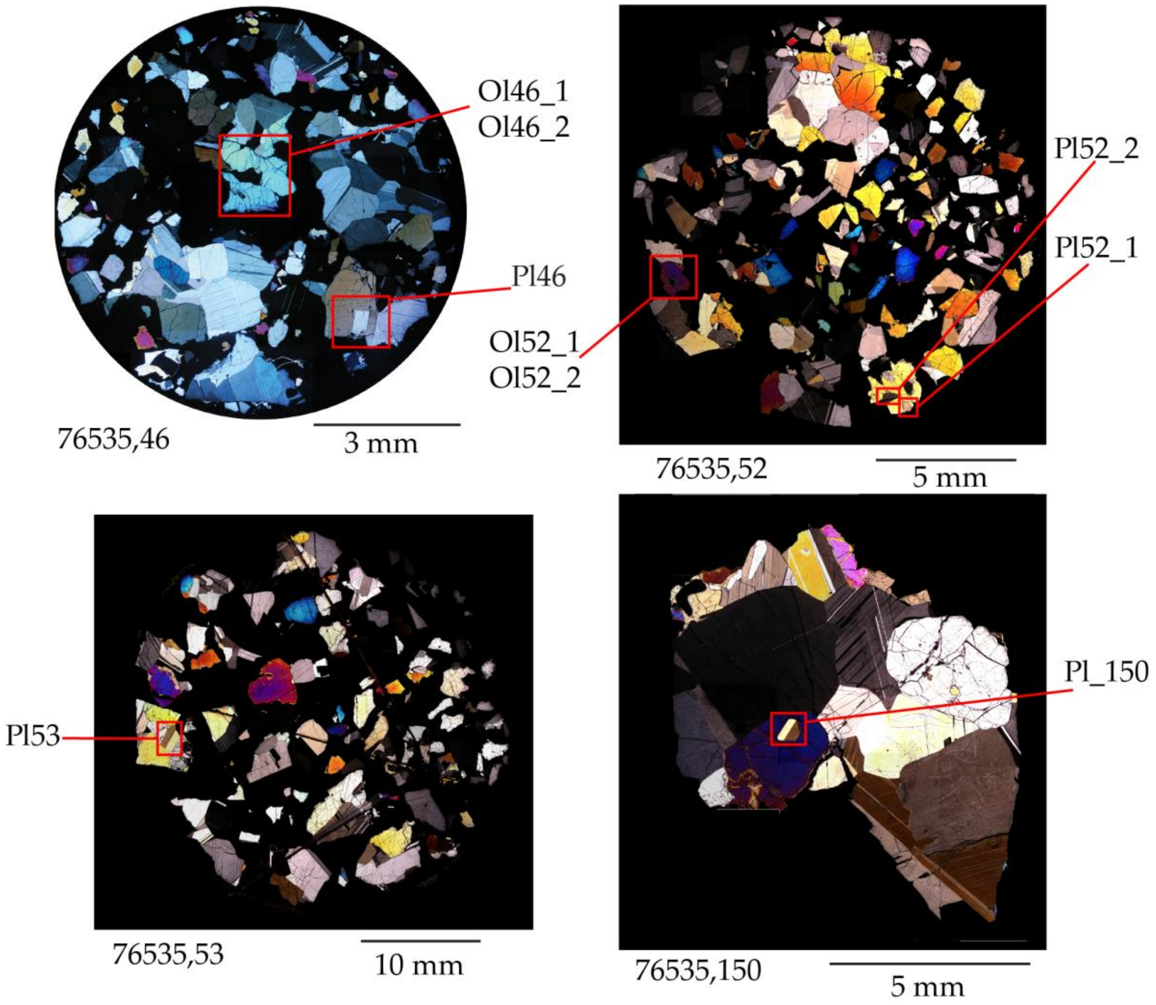

Supplementary Figure 5: Locations of all grains used in this study overlaid on XPL images of slides.

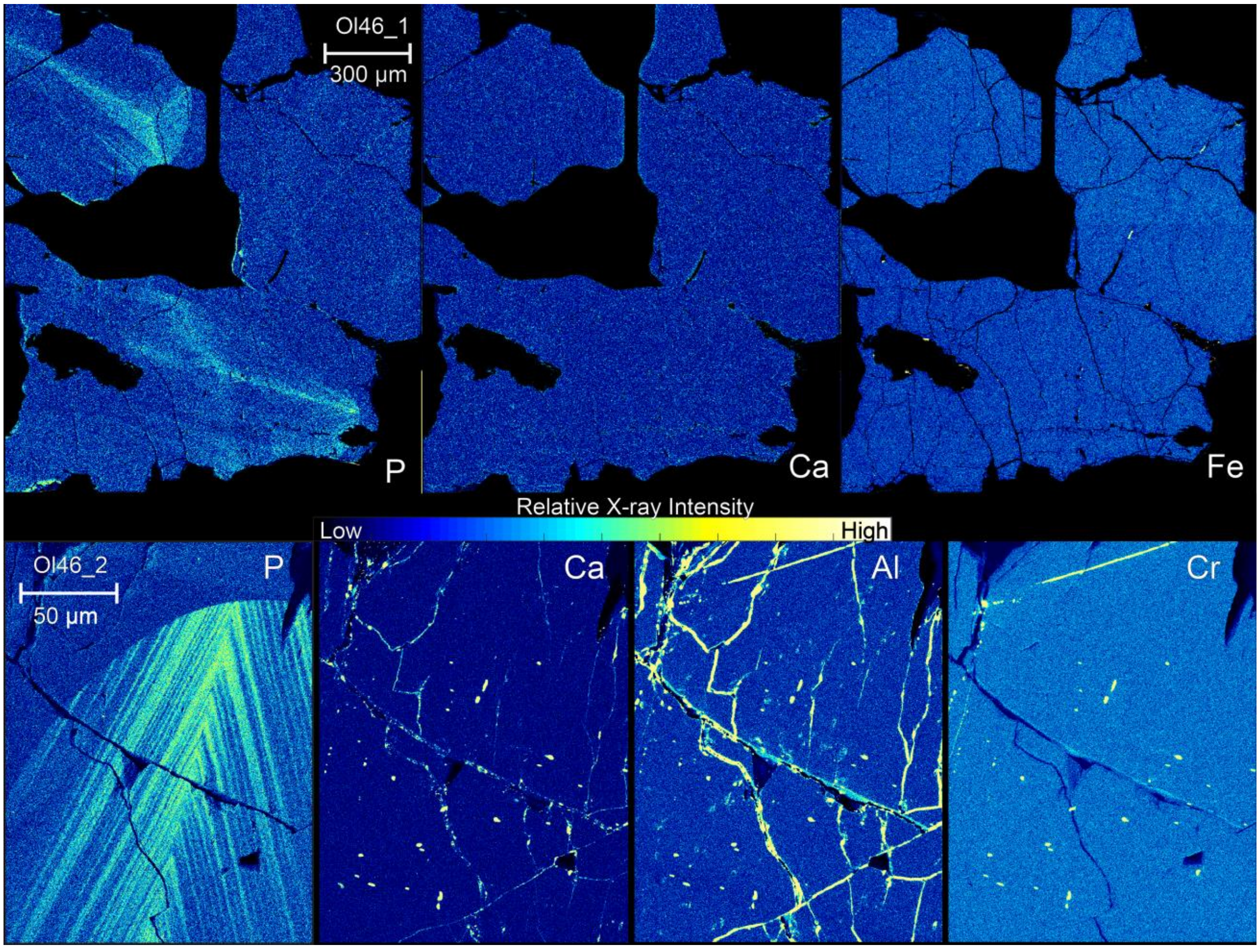

Supplementary Figure 6: X-ray compositional maps of olivine in slide , 46. Though sharp elemental truncation is observed in phosphorus, all other elements are relatively homogeneous within a given olivine grain.

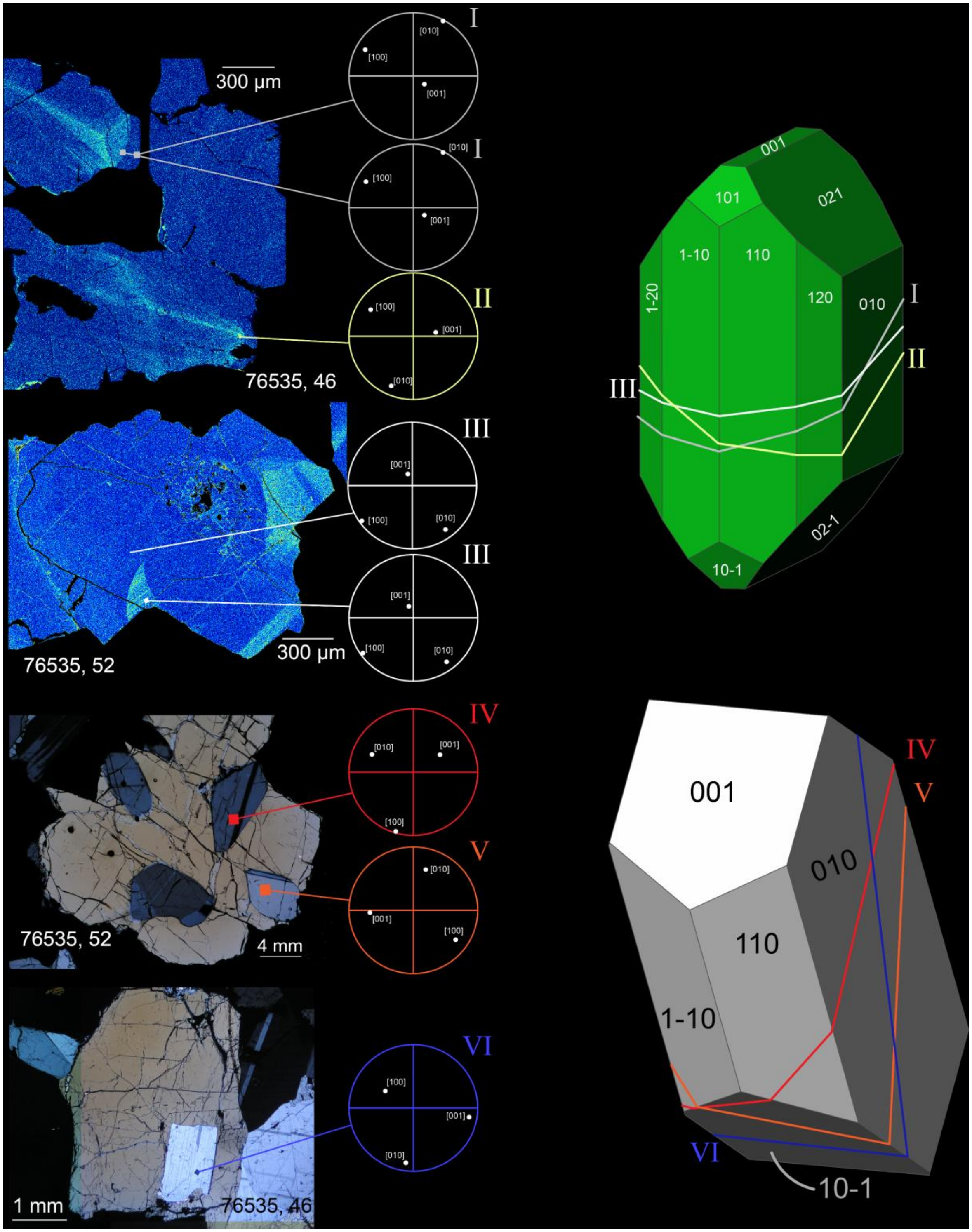

Supplementary Figure 7: Orientations of relevant grains as determined by EBSD. Note that orientations are constant on either side of the truncations in P lamellae. 3D models of each crystal produced in SHAPE v 7.4

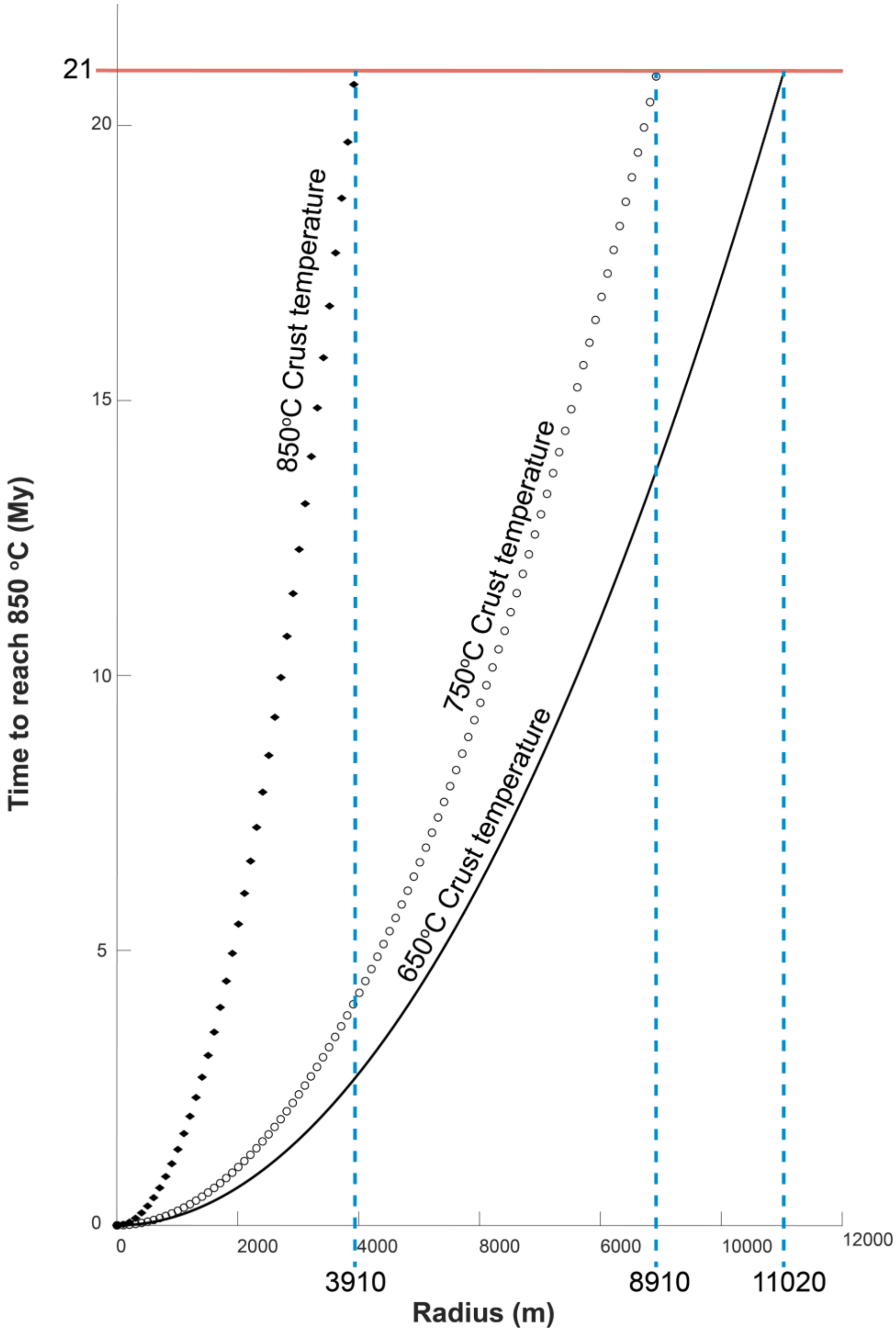

Supplementary Figure 8: Maximum magma chamber radius for a spherical body to cool to 850ºC and still preserve all heterogeneities reported in this study. See the Methods section for details.

Supplementary Table 1: Parent melt composition used for troctolite 76535.

Oxide	SiO_2	TiO_2	Al_2O_3	Cr_2O_3	FeO_T	MnO	MgO	CaO	Na_2O	K_2O	P_2O	Total
O'Sullivan&Neal[1]	46-52	--	19-22	--	8-10	--	10-13	10-12	--	--	--	93-109
Sonzogni&Treiman[2]	57.92	0.39	17.52	0.06	2.89	0.06	10.08	10.70	0.29	0.07	0	99.98
This study	47.16	0.39	19.16	0.06	8.00	0.10	13.01	11.79	0.19	0.07	0.07	100.00

Supplementary Table 2: Standard analyses used for olivine measurements. bd= below detection (<30 ppm).

	A-99 Glass (NMNH 113498-1, n=6)					Springwater olivine (USNM 2566, n=6)				
Oxide	P_2O_5	SiO_2	MgO	FeO_T	Total	P_2O_5	SiO_2	MgO	FeO_t	Total
Mean (Wt %)	0.39	47.93	5.06	13.18	66.56	bd	38.97	44.31	16.62	99.89
Standard Deviation	0.00	0.17	0.02	0.06	0.26	-	0.11	0.09	0.03	0.22
Relative Deviation	0.01	0.00	0.00	0.00	0.02	-	0.00	0.00	0.00	1.13
Published[3] (Wt%)	0.38	50.94	5.08	13.49	69.89	-	38.95	43.58	16.62	99.15
Avg/Published	1.03	0.94	1.00	0.98	-	-	1.00	1.02	1.00	-

Supplementary Table 3: Standard analyses used for plagioclase measurements.

	Anorthite standard (NMNH 137041, n=17)							
Oxide	SiO_2	Al_2O_3	FeO_T	MgO	CaO	Na_2O	K_2O	Total
Mean (Wt %)	43.68	35.85	0.46	0.04	19.06	0.53	0.01	99.63
Standard Deviation	0.29	0.12	0.01	0.00	0.08	0.01	0.01	0.53
Relative Deviation	0.01	0.00	0.03	0.08	0.00	0.03	0.56	0.72
Published[3](Wt %)	44.00	36.03	0.62	0.02	19.09	0.53	0.03	100.32
Avg/Published	0.99	1.00	0.75	1.95	1.00	0.99	0.30	-

Supplementary Table 4: Profile from Pl46 (Figure 2)

Distance(um)	SiO2	Al_2O_3	Cr_2O_3	FeO_T	NiO	MgO	CaO	Na_2O	K_2O	total
0.00	40.22	0.60	0.04	11.71	0.00	46.82	0.27	0.00	0.00	99.66
15.18	44.20	36.21	0.01	0.13	0.01	0.09	19.47	0.41	0.06	100.59
30.64	44.14	35.93	0.00	0.10	0.00	0.10	19.43	0.44	0.07	100.20
46.03	44.30	36.07	0.00	0.08	0.00	0.09	19.47	0.46	0.07	100.53
61.30	44.38	36.11	0.00	0.07	0.00	0.09	19.53	0.43	0.07	100.67
76.79	44.24	36.04	0.00	0.08	0.01	0.15	19.49	0.46	0.07	100.54
92.33	44.22	36.02	0.00	0.05	0.01	0.08	19.42	0.45	0.07	100.31
107.51	44.32	36.12	0.01	0.05	0.00	0.08	19.51	0.44	0.07	100.60
123.09	44.30	36.20	0.00	0.05	0.01	0.09	19.55	0.44	0.07	100.70
138.31	44.25	36.01	0.01	0.06	0.00	0.13	19.56	0.43	0.06	100.52
153.62	44.19	36.16	0.01	0.05	0.01	0.08	19.57	0.40	0.06	100.54
169.16	44.13	36.15	0.00	0.05	0.00	0.07	19.50	0.43	0.06	100.39
184.47	44.08	36.18	0.00	0.05	0.01	0.09	19.56	0.43	0.07	100.45
199.74	44.07	35.88	0.00	0.19	0.00	0.57	19.35	0.42	0.06	100.54
215.19	44.05	36.24	0.00	0.06	0.00	0.11	19.53	0.42	0.06	100.47
230.46	44.11	36.27	0.00	0.03	0.01	0.06	19.60	0.42	0.07	100.57
245.86	44.11	36.08	0.01	0.06	0.00	0.22	19.59	0.42	0.06	100.57
261.48	44.09	36.18	0.00	0.10	0.01	0.29	19.60	0.40	0.06	100.72
276.66	44.00	36.27	0.01	0.04	0.01	0.06	19.64	0.42	0.06	100.52
292.24	43.98	36.22	0.00	0.08	0.01	0.22	19.52	0.41	0.06	100.50
307.51	43.90	36.24	0.00	0.07	0.01	0.19	19.54	0.42	0.06	100.44
322.78	43.93	36.30	0.01	0.05	0.01	0.08	19.56	0.41	0.06	100.39
338.32	43.88	36.31	0.00	0.04	0.02	0.06	19.63	0.41	0.06	100.41
353.63	43.86	36.10	0.01	0.08	0.00	0.25	19.55	0.41	0.06	100.32
368.94	43.83	36.06	0.01	0.05	0.00	0.19	19.68	0.39	0.06	100.27
384.34	43.91	36.28	0.00	0.03	0.00	0.06	19.65	0.42	0.06	100.41
399.79	40.88	34.15	0.00	0.05	0.00	0.12	18.25	0.40	0.06	93.92
415.02	43.95	36.01	0.04	0.16	0.00	0.38	19.45	0.39	0.06	100.45
430.46	43.79	36.15	0.01	0.05	0.01	0.08	19.63	0.39	0.06	100.17
445.86	43.98	36.21	0.00	0.05	0.00	0.10	19.65	0.38	0.06	100.43
461.17	43.95	36.22	0.01	0.05	0.00	0.09	19.63	0.39	0.06	100.39
476.62	43.46	36.07	0.00	0.03	0.00	0.05	19.49	0.39	0.05	99.54
491.98	43.79	36.21	0.00	0.04	0.00	0.07	19.66	0.40	0.06	100.23
507.42	43.90	36.07	0.00	0.05	0.01	0.10	19.63	0.38	0.06	100.21
522.78	43.91	36.13	0.00	0.06	0.00	0.12	19.65	0.38	0.06	100.32
538.14	43.94	36.01	0.01	0.06	0.00	0.12	19.66	0.39	0.06	100.26
553.60	43.89	36.03	0.01	0.06	0.00	0.12	19.63	0.40	0.06	100.19
568.90	43.91	36.02	0.00	0.06	0.01	0.12	19.70	0.38	0.06	100.27
584.26	43.96	36.05	0.00	0.05	0.01	0.10	19.60	0.40	0.06	100.23
599.66	43.90	36.01	0.00	0.06	0.01	0.11	19.54	0.42	0.06	100.12
615.11	43.91	35.90	0.00	0.06	0.00	0.12	19.59	0.42	0.06	100.07
630.51	44.03	35.86	0.00	0.07	0.00	0.13	19.57	0.41	0.06	100.13
645.87	44.05	35.73	0.12	0.11	0.00	0.20	19.51	0.45	0.07	100.24
661.22	44.10	35.75	0.00	0.07	0.01	0.12	19.43	0.45	0.07	99.98
676.63	44.13	35.77	0.00	0.07	0.00	0.14	19.47	0.44	0.07	100.09
691.94	44.22	35.82	0.00	0.07	0.02	0.13	19.42	0.45	0.07	100.19
707.34	43.81	35.73	0.00	0.08	0.00	0.14	19.44	0.44	0.07	99.70
722.70	43.81	35.94	0.01	0.08	0.00	0.09	19.51	0.43	0.07	99.94
738.10	43.33	35.95	0.00	0.10	0.00	0.06	19.38	0.40	0.05	99.27

Supplementary Table 5: Profile from pl52_1 (Supplementary Figure 3).

Distance (um)	Na2O WT%	SiO2 WT%	Al2O3 WT%	CaO WT%	FeO_T WT%	MgO WT%	K2O WT%	Total
0.00	0.33	43.15	35.93	19.51	0.03	0.06	0.05	99.05
5.63	0.35	43.44	36.04	19.40	0.04	0.06	0.05	99.39
11.11	0.36	43.94	36.08	19.49	0.04	0.07	0.04	100.02
16.71	0.37	43.38	36.09	19.41	0.03	0.06	0.04	99.39
22.28	0.36	43.56	36.14	19.42	0.05	0.07	0.05	99.65
27.76	0.35	43.58	35.36	19.42	0.04	0.23	0.05	99.03
33.16	0.24	45.03	30.19	20.54	0.32	3.14	0.03	99.50
38.78	0.20	47.03	24.58	21.48	0.64	6.01	0.03	99.96
44.27	0.34	43.56	36.08	19.47	0.04	0.07	0.04	99.61
49.75	0.37	43.47	36.12	19.43	0.03	0.06	0.04	99.53
55.31	0.37	43.43	36.09	19.38	0.04	0.07	0.04	99.40
61.00	0.37	43.47	35.90	19.43	0.05	0.08	0.04	99.34
66.40	0.37	43.40	36.00	19.49	0.03	0.08	0.04	99.43
71.88	0.36	43.36	36.00	19.41	0.04	0.08	0.05	99.31
77.56	0.35	43.35	35.95	19.40	0.04	0.09	0.04	99.22
83.12	0.35	43.12	35.95	19.46	0.03	0.09	0.04	99.04
88.61	0.35	43.26	35.90	19.38	0.05	0.10	0.05	99.08
94.21	0.34	43.49	35.92	19.45	0.06	0.11	0.05	99.41
99.63	0.33	43.22	35.89	19.47	0.05	0.10	0.04	99.10
105.18	0.34	43.06	35.88	19.52	0.03	0.10	0.04	98.98
110.66	0.34	43.09	35.88	19.40	0.04	0.11	0.04	98.90
116.27	0.34	43.24	35.80	19.35	0.04	0.11	0.05	98.92
121.83	0.34	43.19	35.80	19.39	0.04	0.11	0.04	98.91
127.37	0.34	42.75	35.79	19.35	0.05	0.10	0.03	98.41
133.00	0.35	43.02	35.68	19.33	0.03	0.10	0.05	98.56
138.40	0.33	42.73	35.82	19.44	0.05	0.11	0.04	98.51
144.02	0.35	43.62	36.30	19.34	0.06	0.11	0.04	99.81
149.38	0.34	42.57	35.40	19.21	0.05	0.10	0.04	97.72
154.98	0.33	42.89	35.75	19.38	0.05	0.12	0.03	98.55
160.56	0.33	32.93	32.20	15.48	0.20	0.17	0.11	81.42
166.11	0.34	42.19	35.48	19.25	0.05	0.12	0.05	97.48
171.65	0.35	42.82	35.72	19.41	0.04	0.12	0.04	98.50
177.19	0.33	43.04	35.79	19.36	0.05	0.11	0.05	98.72
182.67	0.33	42.92	35.94	19.42	0.04	0.12	0.05	98.82
188.24	0.36	43.19	35.76	19.45	0.04	0.12	0.04	98.96
193.78	0.33	43.29	35.89	19.36	0.04	0.12	0.05	99.07
199.40	0.32	43.46	36.02	19.32	0.05	0.12	0.04	99.32
204.87	0.33	43.25	35.92	19.38	0.04	0.11	0.04	99.08
210.51	0.35	43.40	36.02	19.42	0.05	0.11	0.05	99.40
215.99	0.35	42.87	35.83	19.31	0.05	0.12	0.04	98.56
221.46	0.34	43.32	35.92	19.41	0.04	0.12	0.04	99.19
227.00	0.34	43.42	35.91	19.39	0.05	0.11	0.04	99.26

Supplementary Table 6: Profile from Ol52

Distance (um)	P2O5 WT%	SiO2 WT%	MgO WT%	FeO_T WT%	Total
0.00	0.03	40.27	48.44	12.09	100.82
4.96	0.02	40.32	48.40	12.13	100.87
10.13	0.02	40.37	48.44	12.07	100.89
15.11	0.04	40.38	48.47	12.08	100.97
20.07	0.04	40.38	48.46	12.12	100.99
25.12	0.04	40.38	48.54	12.03	100.99
30.20	0.03	40.39	48.51	12.15	101.07
35.09	0.03	40.37	48.49	12.19	101.08
40.25	0.03	40.34	48.34	12.20	100.92
45.05	0.03	40.38	48.36	12.29	101.05
50.10	0.03	40.37	48.40	12.21	101.00
55.15	0.06	40.36	48.40	12.27	101.08
60.21	0.05	40.31	48.30	12.23	100.89
65.12	0.03	40.35	48.40	12.16	100.93
70.18	0.03	40.35	48.46	12.21	101.05
75.03	0.03	40.36	48.49	12.21	101.08
80.00	0.03	40.35	48.48	12.21	101.07
85.18	0.04	40.37	48.49	12.22	101.13
90.25	0.03	40.41	48.45	12.20	101.10
95.12	0.03	40.72	48.03	12.19	100.98
100.20	0.02	40.18	48.27	12.18	100.66
105.27	0.04	40.24	48.47	12.21	100.97
110.12	0.03	40.28	48.50	12.25	101.06
115.20	0.05	40.26	48.44	12.16	100.92
120.28	0.04	40.26	48.42	12.18	100.91
125.24	0.05	40.30	48.45	12.18	100.97
130.21	0.06	40.23	48.43	12.22	100.93
135.19	0.05	40.20	48.49	12.20	100.94
140.25	0.05	40.19	48.42	12.19	100.86
145.22	0.06	40.15	48.41	12.19	100.82
150.18	0.05	40.19	48.39	12.20	100.83
155.17	0.05	40.21	48.42	12.17	100.85
160.21	0.04	40.23	48.37	12.17	100.80
165.21	0.03	40.22	48.33	12.26	100.84
170.16	0.03	40.23	48.32	12.15	100.74
175.15	0.03	40.22	48.31	12.21	100.78
180.29	0.05	40.18	48.32	12.18	100.73
185.38	0.05	40.17	48.35	12.15	100.71
190.16	0.04	40.16	48.33	12.27	100.79
195.22	0.01	40.14	48.32	12.25	100.70
200.27	0.00	40.15	48.29	12.23	100.67
205.14	0.00	40.20	48.39	12.16	100.75

Supplementary Table 7: Profile for Ol46 with all 5 spectrometers analyzing phosphorus. Analysis assumed MgO, FeO_t, and SiO_2 matching average olivine composition in Ol52 (Supplementary Table 6).

Distance (um)	P2O5 WT%	SiO2 WT%	MgO WT%	FeO_t WT%	Total
0.000	0.007	40.29	48.40	12.19	100.88
1.475	0.009	40.29	48.40	12.19	100.88
2.905	0.006	40.29	48.40	12.19	100.88
4.471	0.009	40.29	48.40	12.19	100.88
5.996	0.007	40.29	48.40	12.19	100.88
7.474	0.008	40.29	48.40	12.19	100.88
8.959	0.022	40.29	48.40	12.19	100.90
10.623	0.022	40.29	48.40	12.19	100.90
12.012	0.055	40.29	48.40	12.19	100.93
13.533	0.050	40.29	48.40	12.19	100.92
15.011	0.025	40.29	48.40	12.19	100.90
16.539	0.025	40.29	48.40	12.19	100.90
18.155	0.023	40.29	48.40	12.19	100.90
19.585	0.027	40.29	48.40	12.19	100.90
22.231	0.051	40.29	48.40	12.19	100.92
24.011	0.030	40.29	48.40	12.19	100.90
25.538	0.031	40.29	48.40	12.19	100.90
26.876	0.055	40.29	48.40	12.19	100.93
28.667	0.039	40.29	48.40	12.19	100.91
30.055	0.024	40.29	48.40	12.19	100.90
31.541	0.024	40.29	48.40	12.19	100.90
33.068	0.019	40.29	48.40	12.19	100.89
34.681	0.022	40.29	48.40	12.19	100.90
36.076	0.022	40.29	48.40	12.19	100.90
37.600	0.023	40.29	48.40	12.19	100.90
39.124	0.021	40.29	48.40	12.19	100.90
40.512	0.027	40.29	48.40	12.19	100.90
42.190	0.057	40.29	48.40	12.19	100.93
43.631	0.078	40.29	48.40	12.19	100.95
45.109	0.024	40.29	48.40	12.19	100.90
46.724	0.027	40.29	48.40	12.19	100.90
48.199	0.037	40.29	48.40	12.19	100.91
49.677	0.082	40.29	48.40	12.19	100.96
51.243	0.087	40.29	48.40	12.19	100.96
52.767	0.054	40.29	48.40	12.19	100.93
54.067	0.043	40.29	48.40	12.19	100.92
55.633	0.054	40.29	48.40	12.19	100.93
57.249	0.064	40.29	48.40	12.19	100.94
58.552	0.027	40.29	48.40	12.19	100.90
60.340	0.065	40.29	48.40	12.19	100.94
61.773	0.066	40.29	48.40	12.19	100.94
63.114	0.085	40.29	48.40	12.19	100.96
64.635	0.093	40.29	48.40	12.19	100.97
66.163	0.075	40.29	48.40	12.19	100.95
67.687	0.090	40.29	48.40	12.19	100.96
69.208	0.072	40.29	48.40	12.19	100.95
70.736	0.093	40.29	48.40	12.19	100.97
72.128	0.105	40.29	48.40	12.19	100.98
73.744	0.071	40.29	48.40	12.19	100.95
75.306	0.035	40.29	48.40	12.19	100.91
76.559	0.083	40.29	48.40	12.19	100.96
78.271	0.047	40.29	48.40	12.19	100.92
79.656	0.058	40.29	48.40	12.19	100.93
81.269	0.062	40.29	48.40	12.19	100.94
82.747	0.027	40.29	48.40	12.19	100.90
84.363	0.043	40.29	48.40	12.19	100.92
85.703	0.072	40.29	48.40	12.19	100.95
87.316	0.040	40.29	48.40	12.19	100.91
88.711	0.082	40.29	48.40	12.19	100.96
90.236	0.081	40.29	48.40	12.19	100.95
91.891	0.030	40.29	48.40	12.19	100.90
93.232	0.066	40.29	48.40	12.19	100.94
94.798	0.080	40.29	48.40	12.19	100.95
96.320	0.074	40.29	48.40	12.19	100.95
97.711	0.043	40.29	48.40	12.19	100.92
99.052	0.052	40.29	48.40	12.19	100.93
100.704	0.073	40.29	48.40	12.19	100.95
102.407	0.053	40.29	48.40	12.19	100.93
103.707	0.088	40.29	48.40	12.19	100.96
105.362	0.106	40.29	48.40	12.19	100.98
106.802	0.103	40.29	48.40	12.19	100.98
108.146	0.113	40.29	48.40	12.19	100.99
109.855	0.104	40.29	48.40	12.19	100.98
111.299	0.077	40.29	48.40	12.19	100.95
112.820	0.088	40.29	48.40	12.19	100.96
114.344	0.075	40.29	48.40	12.19	100.95
115.869	0.073	40.29	48.40	12.19	100.95

References – Supplementary Materials

1. Welsch, B., Hammer, J. & Hellebrand, E. Phosphorus zoning reveals dendritic architecture of olivine. *Geology* **42**, 867–870 (2014).

2. Sonzogni, P. Y. & Treiman, A. H. Parent magma compositions of the lunar highlands Mg-suite rocks: a melt inclusion perspective. *Lunar Planet. Sci.* **XLVI**, Abstract-2671 (2015).

3. Jarosewich, E., Nelen, J. A. & Norberg, J. A. Reference Samples for Electron Microprobe Analysis. *Geostandards Newsletter* **4**, 43–47 (1980).
