# Supplementary material for: Chemical heterogeneities reveal early rapid cooling of Apollo Troctolite 76535
Source: Nat Commun. 2021 Dec 14;12:7054. doi: 10.1038/s41467-021-26841-4 (PMC8671448; doi:10.1038/s41467-021-26841-4)
Supplement: Supplementary file 3 — Description of Additional Supplementary Files [file 41467_2021_26841_MOESM3_ESM.pdf]

Description of Additional Supplementary Files

File name: Supplementary Movie 1

Description: A video showing an example of finding a, and b for a cooling path T=T0-a*log(b*t+1) in Ol46_2. See methods section for more details
