# Supplementary figures and images for: Chemical heterogeneities reveal early rapid cooling of Apollo Troctolite 76535
Source: Nat Commun. 2021 Dec 14;12:7054. doi: 10.1038/s41467-021-26841-4 (PMC8671448; doi:10.1038/s41467-021-26841-4)

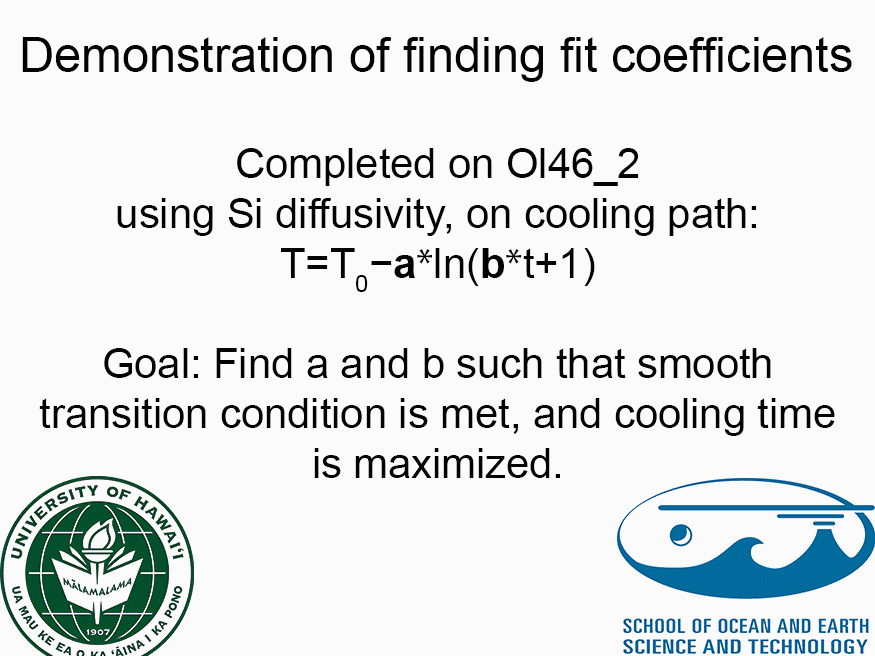

Supplement: Supplementary file 4 — Supplementary Video 1 [file 41467_2021_26841_MOESM4_ESM.gif]
